# Epidural Metalloma 12 Years After Spinal Fusion for Adolescent Idiopathic Scoliosis: A Case Report

**DOI:** 10.1007/s43465-025-01425-2

**Published:** 2025-06-02

**Authors:** Janez Mohar, René Mihalič, Dejan Čeleš

**Affiliations:** 1 Department of Spine Surgery, Valdoltra Orthopaedic Hospital, Jadranska Cesta 31, 6280 Ankaran, Slovenia; 2 Chair of Orthopaedics, Faculty of Medicine, University of Ljubljana, Zaloška Cesta 9, 1000 Ljubljana, Slovenia

**Keywords:** Scoliosis, Pseudoarthrosis, Corrosion, Neoplasms, Return to sport

## Abstract

**Introduction:**

The occurrence of metallosis after spine surgery is a rare phenomenon. The accumulation and deposition of metallic nanoparticles, especially cobalt and chromium, elicit a local inflammatory response by modulating the expression of cytokines, ultimately manifesting as a pseudotumor. This process is considered an aseptic lymphocyte-dominated vasculitis-associated lesion or adverse local tissue reaction (ALTR).

**Methods:**

We present a case of metalloma that developed 12 years after surgery for adolescent idiopathic scoliosis (AIS).

**Results:**

After a piecemeal decompression of the pseudocyst, additional internal fixation instrumentation, and a pseudarthrosis revision with bone transplantation, the patient regained all prior flexibility and remained pain free.

**Conclusion:**

We speculate that the proposed mechanism of instrumentation failure in our patient was over-aggressive postoperative exercise with pseudoarthrosis and metal-on-metal corrosion, leading to ALTR in the spinal canal.

## Introduction

Postoperative metallosis of the spine is a rare phenomenon, especially after surgery for adolescent idiopathic scoliosis (AIS). The accumulation and deposition of 25–50 nm metallic nanoparticles, including cobalt and chromium, elicit a local inflammatory response, owing to cytokine expression modulation secondary to abnormal wear. Local manifestations of this response, known as aseptic lymphocyte-dominated vasculitis-associated lesions, include bone and/or tissue necrosis as well as pseudotumor formation. Bio-tribocorrosion is a phenomenon involving the degradation of materials, owing to the corrosive and tribological characteristics of the biological environment. Tribology involves micromovements at the bone–metal interface of spinal implants, resulting in corrosion of the metal. The loosening of the metal-on-metal junction, which is considered another site of primary corrosion, can accelerate this process. If the abnormal micromovements and/or reaction continue, this cycle can result in the development of a metalloma, which is an inflammatory granulation tissue mass, or an adverse local tissue reaction (ALTR). It is thought; therefore, that metallosis can be prevented by ensuring the rigid fixation of the fused complex [1]. Unlike with arthroplasty, there should be no movement after spinal fusion. Advances in modern instrumentation have resulted in the improved biomechanical stability of surgical constructs and a more stable environment in which bony fusion can occur postspinal surgery [2]. However, metallosis rarely occurs in patients who have undergone spinal fusion surgery, suggesting that the cause of this phenomenon is more complex than simple tribology. To help improve our understanding of this condition, we present a case of metallosis that developed 12 years post-AIS correction and fusion, including details of the patient diagnostics and management and a proposed causative factor. This manuscript was prepared using the case report (CARE) guidelines.

## Case Presentation

A 25-year-old man presented to our outpatient clinic with complaints of lower back pain radiating to his right lower extremity. He repeatedly experienced neurogenic claudication and could only walk approximately 500 m without stopping. The patient was 12 years postspinal fusion with a hybrid construct from thoracic vertebra 5 (T5) to lumbar vertebra 4 (L4) that involved pedicle screws in the lumbar vertebrae and hooks in the thoracic vertebrae (CD Horizon Legacy; Medtronic, Minneapolis, MN, USA)**.** From the time of his surgery until 1 month prior to his presentation, the patient had been asymptomatic, followed-up regularly, and very physically active. The patient was fully ambulatory when he arrived at our clinic and did not present with any gait abnormalities.

**Fig. 1:**
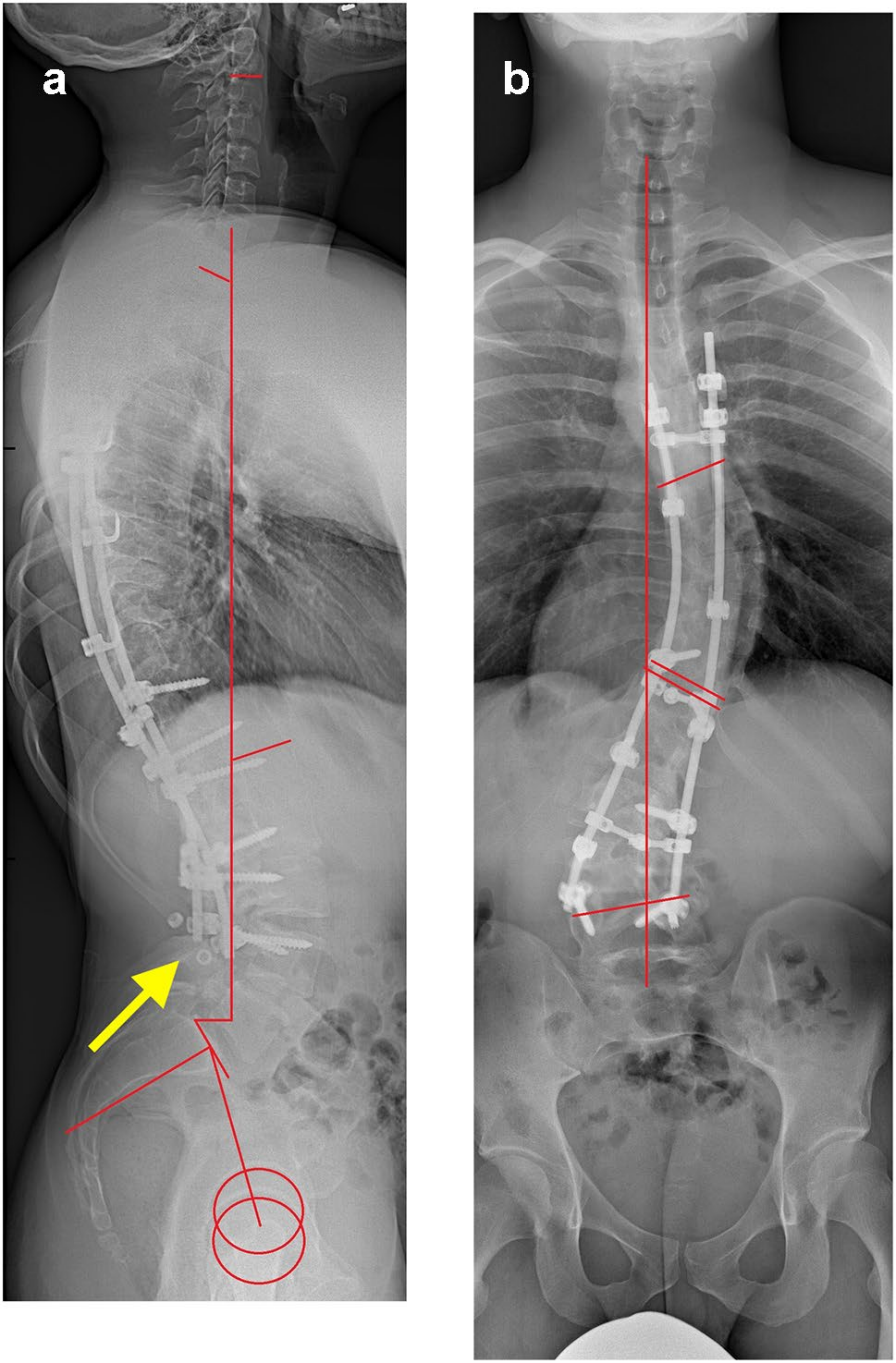
Anteroposterior and lateral radiographs of the patient’s spine. Thoracic Cobb angle, 47°; lumbar Cobb angle, 37°; central sacral vertical line, sagittal vertical axis (19 mm); L1–S1 lumbar lordosis, 70°; pelvic tilt, 12°; sacral slope, 52°; and T5–T12 thoracic kyphosis, 43° (red lines). Dislodged set screws are visible on the lateral projection (yellow arrow)

**Fig. 2:**
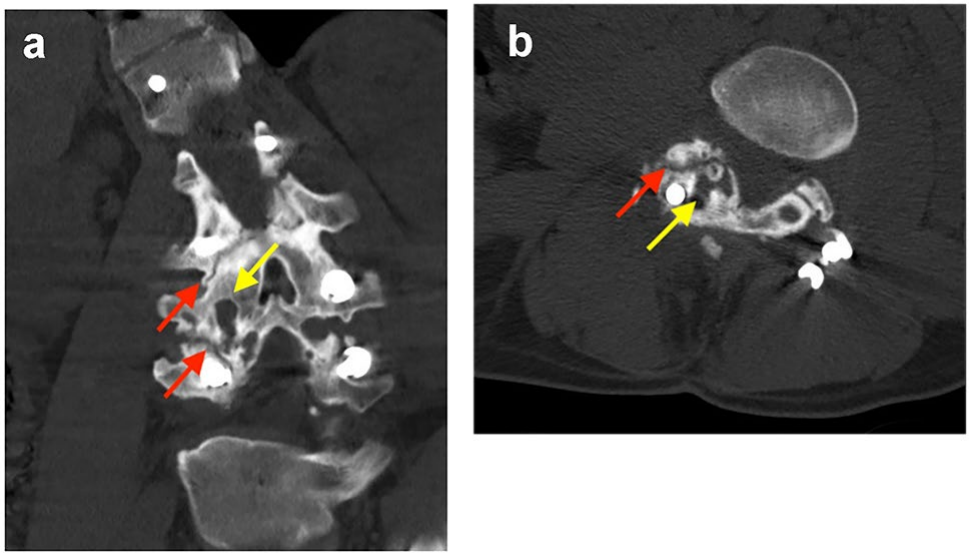
Coronal and axial computed tomography images showing pseudarthrosis (red arrow) and a bone cyst (yellow arrow)

**Fig. 3:**
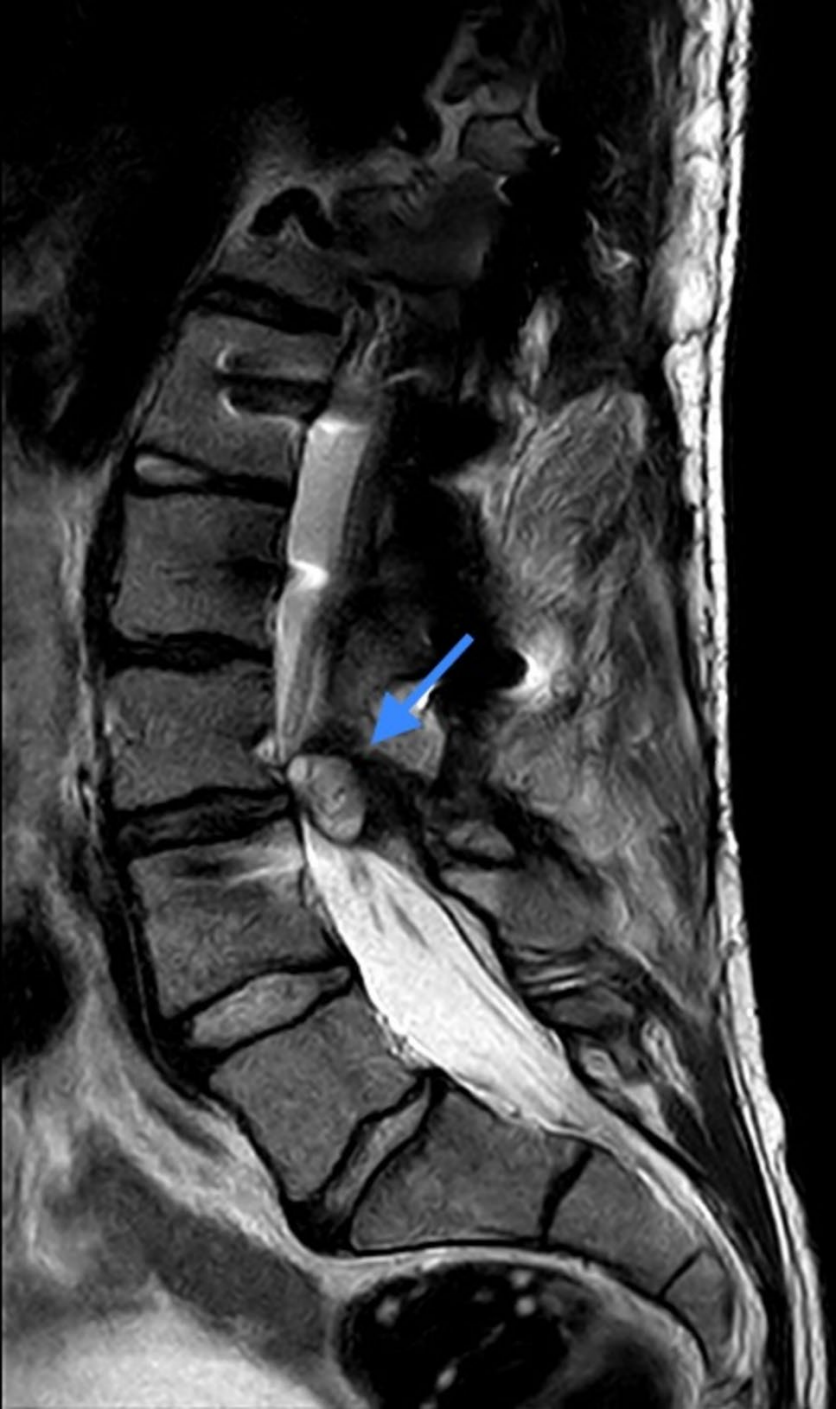
Sagittal magnetic resonance imaging showing lobulated epidural cystic formation (blue arrow)

The results of provocative diagnostic tests for radiculopathy were negative, whereas laboratory test results demonstrated an elevated leukocyte count (12.4 × 10⁹/L) and serum monocyte fraction (1 × 10⁹/L). Anteroposterior and lateral radiographs of the spine exhibited three unlocked and dislodged pedicle set screws at L3 left and L4 bilateral (Fig. 1), whereas computed tomography showed pseudarthrosis and cystic bone formations at L3/4 with no signs of pedicle screw loosening (Fig. 2). Magnetic resonance imaging (MRI) revealed a lobulated, cystic, fluid-filled formation in the spinal canal at the L3/4 disc, predominantly on the right side (Fig. 3).

We performed an open surgical exploration of the lesion with a posterior midline approach to visualize the bony structures. The unlocked and dislodged set screws were replaced and the construct was refixed to the lowest two instrumented vertebrae (L4). A wide central laminotomy at the L3/4 segment revealed the dural sac as well as a tumorous cystic formation. The cysts were removed using a piecemeal technique and the dural sac was fully decompressed. The site of pseudarthrosis was decorticated with local transplantation of a free bone graft.

**Fig. 4:**
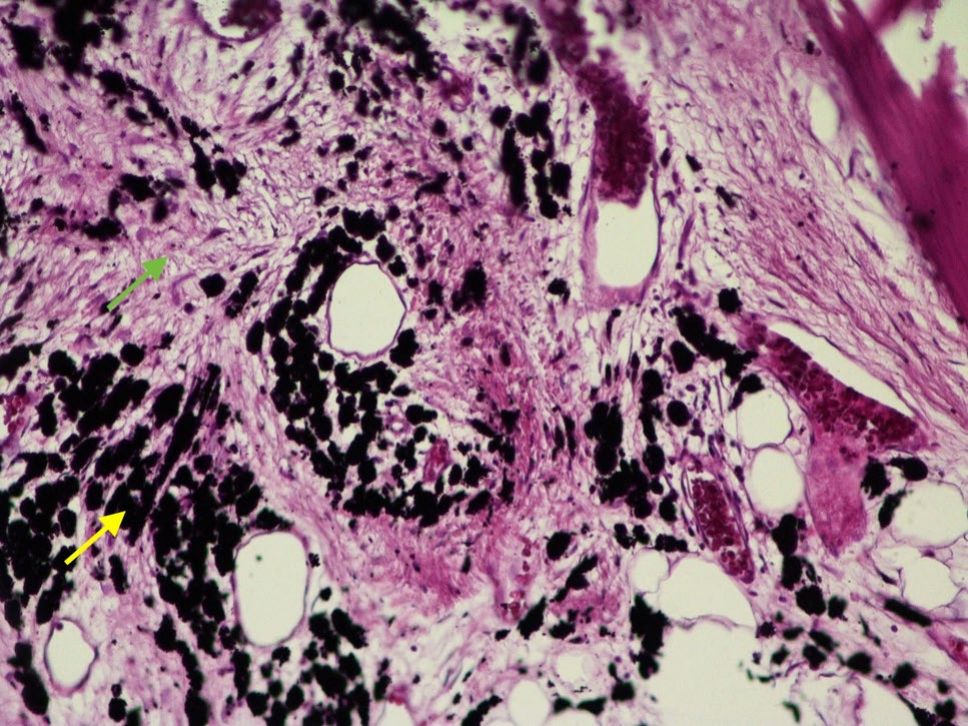
Histopathological image of the metalloma. Microscopic image of connective tissue (green arrow) with black metal debris within macrophages (yellow arrow); hematoxylin and eosin staining, 400 × magnification

The postoperative course was uneventful, with follow-up MRI showing the complete resection of the cystic lesion. Microscopic histopathology revealed pseudocystic spaces with no epithelial lining that were filled with fibrous tissue and metallic particles, consistent with a diagnosis of epidural ALTR (Fig. 4). At the patient’s two-year post-revision follow-up, he was asymptomatic with a stable T5–L4 construct.

## Discussion

We have described herein the third reported case of postsurgical metallosis to occur after the treatment of AIS [2, 3]. Richman et al. [2] described a peculiar case of metallosis with no appreciable pseudarthrosis, implant loosening, or infection, whereas Beguiristain et al. [3] observed the colonisation of *Propionibacterium acnes* and Botolin et al. [4] noted loosening of the implant at the bone-on-metal junction, a known cause of corrosion. To the best of our knowledge, this is the first case to demonstrate loosening at the metal-on-metal junction as a causative factor for corrosion and ALTR following AIS surgery. Because the patient was physically active after his initial procedure, we speculate that an insufficient fusion mass formed, eventually resulting in implant failure.

The guidelines for returning to sport after AIS correction have yet to be established. Lehman et al. [5] found it appropriate to return to noncontact sports at 6 months and contact sports at 1 year postsurgery. Moreover, although Lehman et al. [5] found that the Spinal Deformity Study Group, with > 10,000 combined AIS cases, reported only one case of construct failure with no catastrophic neurologic injuries, they acknowledged that it usually takes 1–2 years for the fusion mass to fully form. Regardless of the current guidance on when it is appropriate to return to sport, we propose the careful postoperative management of patients with AIS constructs ending in the lower lumbar spine.

## Conclusion

The case presented herein is the first reported case of post-AIS surgery ALTR with metal-on-metal junction failure as a causative factor for corrosion. In this case, a metalloma pseudotumor formed near the site of the implant-to-implant failure, showing the penetrating and destructive nature of metallic particles in a biological environment. Owing to the presence of both unlocked pedicle set screws and pseudarthrosis, we surmised that the patient’s return to sport occurred too early for a solid fusion mass to fully form. 

## Data Availability

All data related to the published case report can be obtained upon reasonable request from the corresponding author.
